# Diet and Immune Function

**DOI:** 10.3390/nu11081933

**Published:** 2019-08-16

**Authors:** Caroline E. Childs, Philip C. Calder, Elizabeth A. Miles

**Affiliations:** 1 Human Development and Health, Faculty of Medicine, University of Southampton, Southampton SO16 6YD, UK; 2 NIHR Southampton Biomedical Research Centre, University Hospital Southampton NHS Foundation Trust and University of Southampton, Southampton SO16 6YD, UK

**Keywords:** nutrition, immunity, macronutrients, micronutrients, microbiome, life course, probiotic, prebiotic, inflammation

## Abstract

A well-functioning immune system is critical for survival. The immune system must be constantly alert, monitoring for signs of invasion or danger. Cells of the immune system must be able to distinguish self from non-self and furthermore discriminate between non-self molecules which are harmful (e.g., those from pathogens) and innocuous non-self molecules (e.g., from food). This Special Issue of Nutrients explores the relationship between diet and nutrients and immune function. In this preface, we outline the key functions of the immune system, and how it interacts with nutrients across the life course, highlighting the work included within this Special Issue. This includes the role of macronutrients, micronutrients, and the gut microbiome in mediating immunological effects. Nutritional modulation of the immune system has applications within the clinical setting, but can also have a role in healthy populations, acting to reduce or delay the onset of immune-mediated chronic diseases. Ongoing research in this field will ultimately lead to a better understanding of the role of diet and nutrients in immune function and will facilitate the use of bespoke nutrition to improve human health.

## 1. Overview of the Immune System

Broadly, cells of the immune system may be divided into those of the innate and those of the adaptive immune response. The innate response is the first response to an invading pathogen. Cells of the innate immune response include phagocytes (e.g., macrophages and monocytes), neutrophils, dendritic cells, mast cells, eosinophils, and others. The innate response is rapid, but not specialised and is generally less effective than the adaptive immune response.

The adaptive immune response has the ability to specifically recognise a pathogen and ‘remember’ it if exposed to it again. T cells are critical in antigen recognition and the co-ordination of the immune response. T cells are present in an array of subtypes that coordinate different types of immune responses. Broadly, they are divided into the cytotoxic T cells (bearing the CD8 receptor), which are involved in direct killing of infected damaged cells and tumour cells, and the T helper cells. T helper (Th) cells bear the CD4 receptor and are important in coordinating the responses of other immune cells. There are a number of subtypes of Th cells, defined by the cytokines they produce. Initial studies identified two subsets, the Th1 cells, which produced interferon gamma (IFN-γ) and interleukin (IL)-2 and were important in antiviral and cellular immune responses, and the Th2 subset producing IL-4, IL-5, and IL-13 and involved in humoral (antibody) and anti-parasitic responses (but also in allergic responses) [1]. It is now apparent that there are a number of other Th subtypes, which do not fall into these categories. This includes Th17 cells, which produce IL-17A, IL-17F, and IL-22 and are important in fighting extracellular pathogens (bacteria and fungi) [2]. There are also T regulatory cells (Treg), which are CD4-bearing T cells vital in maintaining immune tolerance to allow the immune system to ignore non-harmful non-self (such as food, pollen, and environmental antigens such as latex). Thus, the role of T cells is coordinating an appropriate immune response following immune stimulation or challenge.

The other lymphocytes of the adaptive immune system are the B cells, which are responsible for antibody or immunoglobulin (Ig) production. Like T cells, B cells respond specifically to an antigen. They can differentiate into short-lived plasma cells, which produce Igs in the short term, or can become long-lived plasma cells. Igs are pathogen-specific molecules, which help the immune system to recognise and destroy pathogens. The B cells can differentiate into plasma cells, which produce one of five classes of Ig (IgM, IgD, IgG, IgA, and IgE). Each class of Ig has a specialised role [3]. IgM is the first Ig expressed during development, is often found as a multimeric molecule (e.g., pentameric), and can bind an antigen to identify it for destruction by immune cells. IgD is found in low concentrations in the plasma and the specialist role of IgD is not yet clear. IgG is the predominant Ig class and can persist for long periods. It has important roles in antigen labelling, resulting in more effective removal. IgA can be found in the serum (mostly as a monomer) and at mucosal surfaces (normally as a dimer). At the mucosal surface, IgA protects against bacteria and or viruses, preventing infection. IgA also has an important role in neutralising food antigens and helping to maintain immune tolerance to food antigens (preventing the development of food allergy) [4]. IgE has a role in clearance of extracellular parasites (e.g., helminths) but when produced inappropriately to innocuous environmental and food antigens, has an important role in IgE-mediated allergy. B cells go through a process called class switching to set the class of Ig that the plasma cells derived from them will produce. B cell class switching is controlled by the cytokines present, particularly IL-4, IL-6, and IFN-γ secreted from Th cells [5].

T and B cells can specialise to become memory cells, which persist permanently or for very long periods and are able to recognise the antigen if encountered again and elicit a rapid, pathogen-specific immune response.

The effective deployment of the immune system against pathogens or harmful signals and the swift resolution of the immune response is required for survival. The fighting of infection is only one piece of the puzzle. A fulminating immune response is costly in terms of energy expended and results in damage to the host tissues; thus, rapid and complete resolution of an immune response is also key. Cytokines play a role in resolution of immune responses. IL-10, which is produced by a range of immune cells including Tregs, has anti-inflammatory actions including suppressing inflammatory cytokine production [6].

The instigation of an immune response and the activities of the immune cells results in inflammation (seen as redness, swelling, and the feeling of heat and pain), which are signs of the damage to the tissue going on whilst the immune system does its work. This is an expected outcome of an effective immune response. Increasingly there is concern that modern lifestyle changes have resulted in the promotion of ongoing, low-grade, whole-body (systemic) inflammation caused by immune and other cells (e.g., adipocytes, the cells that store lipids in fat tissue) [7]. Such exposures may include diet quality and quantity [8].

## 2. The Role of Nutrition in Immune Function

Adequate and appropriate nutrition is required for all cells to function optimally and this includes the cells in the immune system. An “activated” immune system further increases the demand for energy during periods of infection, with greater basal energy expenditure during fever for example. Thus, optimal nutrition for the best immunological outcomes would be nutrition, which supports the functions of immune cells allowing them to initiate effective responses against pathogens but also to resolve the response rapidly when necessary and to avoid any underlying chronic inflammation. The immune system’s demands for energy and nutrients can be met from exogenous sources i.e., the diet, or if dietary sources are inadequate, from endogenous sources such as body stores. Some micronutrients and dietary components have very specific roles in the development and maintenance of an effective immune system throughout the life course or in reducing chronic inflammation. For example, the amino acid arginine is essential for the generation of nitric oxide by macrophages, and the micronutrients vitamin A and zinc regulate cell division and so are essential for a successful proliferative response within the immune system.

Undernutrition is well understood to impair immune function, whether as a result of food shortages or famines in developing countries, or as a result of malnutrition arising from periods of hospitalisation in developed countries. The extent of impairment that results will depend upon the severity of the deficiency, whether there are nutrient interactions to consider, the presence of infection, and the age of the subject [9]. A single nutrient can also exert multiple diverse immunological effects, such as in the case of vitamin E, where it has a role as both antioxidant, inhibitor of protein kinase C activity, and potentially interacting with enzymes and transport proteins [10]. For some micronutrients, excessive intake can also be associated with impaired immune responses. For example, supplementation with iron can increase morbidity and mortality of those in malaria endemic regions. As well as nutrition having the potential to effectively treat immune deficiencies related to poor intake, there is a great deal of research interest in whether specific nutrient interventions can further enhance immune function in sub-clinical situations, and so prevent the onset of infections or chronic inflammatory diseases.

## 3. Gut-Associated Lymphoid Tissue

The majority of immune cells within the human body are found within the gut-associated lymphoid tissue (GALT), reflecting the importance of this immune tissue in maintaining host health. In ingesting food, we expose ourselves to near constant and massive antigenic stimulation, and our immune system must be able to provide strong and protective immunity against invasive pathogens, while tolerating food proteins and commensal bacteria. In order to achieve this, the GALT contains a variety of sensing and effector immune functions. Dendritic cells and M cells sample the gut content, while plasma B cells within the lamina propria produce IgA, providing protection against pathogenic organisms. Specialised immune regions known as Peyer’s patches, rich in immune cells, allow for communication between immune cells resident within the GALT, propagation of signals to the wider systemic immune system, and the recruitment or efflux of immune cells [11].

Within the gut lumen itself, the human gut microbiome will provide antigens and signals with the potential to interact with resident and systemic immune cells. The composition of the gut microbiome changes over the life course, in response to dietary components, and to environmental factors such as antibiotic exposure. Dietary interventions targeted at the gut microbiome include probiotics and prebiotics. Probiotics are defined as “live microorganisms, which, when consumed in adequate amounts, confer a health benefit of the host” [12] while prebiotics, “a substrate that is selectively utilized by host microorganisms conferring a health benefit” [13], tend to be non-digestible oligosaccharides such as fructo-oligosaccharides and galacto-oligosaccharides. Provision of plant-based diets may enhance the diversity of nutrients that reach the gut microbiome, with the indigestibility of plant cell walls enabling peptides and lipids, which may otherwise have been absorbed in the upper digestive tract to reach the microbiome [14]. There may be circumstances in which immune cells of the GALT come into direct contact with nutrients or gut microbiota, such as in the case of reduced epithelial integrity, or ‘leaky gut’ observed in both acute and chronic gut inflammation [15]. Such changes in gut permeability may be influenced by micronutrient status such as that of vitamin D [16].

A number of nutrients and dietary interventions have demonstrated the capacity to improve measures of gut health or to reduce gut inflammation. Protein hydrolysates have been demonstrated to enhance barrier function and IgA production in animal models, and as a result may have applications for incorporation within hypo-allergenic infant formula and clinical nutrition for those with conditions such as inflammatory bowel disease [17]. Animal models of gut inflammation have identified that providing probiotic bacteria can reduce inflammation, with reductions in proinflammatory Th1 and Th17 cytokines such as IL-17 and IFN-γ, and enhanced production of inflammation resolving cytokine IL-10 [18]. Prebiotics can also enhance barrier function, in addition to their role as substrates for bacterial metabolism [19]. Santiago-Lopez et al. have investigated the effect of fermented milk on a murine model of inflammatory bowel disease [18] and demonstrated a reduction in serum IL-17 and IFN-γ following fermented milk consumption when compared with the control group.

## 4. Immune Function Over the Life course

The developing foetus and neonates have an immature immune system, with poor antibody production and a low proliferative response to challenge. *In utero*, the foetus can gain passive protection from its mother via antibodies, which cross the placenta. This is the basis by which infants in the UK are provided with early protection against whooping cough, with mothers offered vaccination in their third trimester, in order to provide passive immunity to their infants until they reach the age of infant vaccinations. While immature, the foetal immune system can produce antibodies, and allergens can reach the developing foetus, and allergen-specific IgE can be detected in cord blood samples [20]. Another signature of the immaturity of the immune system in early life is the susceptibility of neonates to infections, and the associated higher burden of morbidity and mortality.

The development of the immune system in early life will be influenced by both feeding practices and environmental exposures. Breastfeeding provides further passive immunity to the infant, for example via transfer of antibodies and cytokines. Breast milk components can also stimulate maturation of the gut-associated lymphoid tissue, with breast milk known to be rich in bifidogenic oligosaccharides and to contain its own unique microbiota. Human milk oligosaccharides (HMOs) are synthesised from lactose in the mammary gland, and the specific HMO profile will vary between individuals and across contexts and changes over the time course of lactation [21]. These HMOs have been found to confer health benefits to infants by inhibiting the adhesion of microorganisms to the intestinal mucosa, enhancing the production of short-chain fatty acids by bacteria within the microbiome, and inhibiting inflammation [22]. Other immune active components of breast milk are also likely to be involved in immune system maturation, with studies identifying that the growth factors epidermal growth factor, fibroblast growth factor 21, and transforming growth factor-β2 can change lymphocyte phenotypes in new-born rats when provided as supplements by oral gavage [23].

In infancy, diverse environmental factors will impact upon immune system development; identified factors include pet ownership, antibiotic use, and the timing of introduction of foods [24]. The opportunity for introduction of prebiotic oligosaccharides during the introduction of foods has been explored, with the suggestion that this could provide a unique opportunity to influence the developing microbiome and thereby interact with the developing immune system [19]. These early years of life are a critical period in the development of the immune system, particularly for T cell function, with the thymus maturing and reaching its maximum size relative to body weight in infancy [25].

As we move through the life course towards later life, a decline in immune function is observed among older adults. As was the case in infancy, older adults are more susceptible to infections, and have more serious complications as a result than younger people. This declining immune function is known as immunosenescence and reflects deterioration of both the acquired and innate immune systems [26]. Declining T cell function with age arises from thymic involution and decreased thymic output, resulting in fewer naive T cells and more memory cells in the circulation [27]. Ageing is also associated with increased inflammation in the absence of infection and has been found to predict hospitalisation and death [28]. A number of micronutrient deficiencies have been identified as contributors to such declining immunity, and so may provide opportunities for targeted interventions to restore immune function [29].

## 5. Chronic Systemic Inflammation

Chronic systemic inflammation is a key underlying feature for a range of chronic non-communicable disease conditions such as cardiovascular disease, stroke, and autoimmune disorders such as rheumatoid arthritis. This chronic inflammation is positively correlated with aging and other co-morbidities (e.g., obesity, cardiovascular disease, insulin resistance). Interestingly, in a study in healthy adults, increasing age was found to be a risk factor for chronic systemic inflammation, independent of other risk factors such as body mass index, blood pressure, and blood lipid profiles [30].

The rising worldwide prevalence of obesity in children and adults is of grave concern. Obesity and over nutrition are strongly associated with chronic inflammation, metabolic perturbation, and higher risk for a number of chronic diseases including cardiovascular disease, stroke, type 2 diabetes mellitus, and chronic liver disease. This metabolism-induced inflammation associated with obesity is termed metaflammation, and the Western diet is a known risk factor [31,32]. The Western diet is characterised by a diet high in sugar, trans and saturated fats, but low in complex carbohydrates, fibre, micronutrients, and other bioactive molecules such as polyphenols and omega 3 polyunsaturated fatty acids. The mechanisms by which the Western diet predispose individuals to metaflammation are still under investigation. However, one mechanism which has been reported is the increased uptake of lipopolysaccharide (LPS, a constituent of gram-negative bacterial cells walls), from microbes in the gut because of increased gut leakiness. This LPS is sensed by cells of the innate immune system through toll-like receptor 4 (TLR4). Activation of TLR4 by LPS will induce an inflammatory response by the immune cells. Certain nutrients, notably long-chain omega 3 polyunsaturated fatty acids, can interfere with TLR4 activation and, thus, can ameliorate this inflammatory signal. Rogero et al. describe the relationship between obesity and inflammation and explores the immune pathway for this mechanism and the anti-inflammatory roles of omega 3 fatty acids in this process [33].

Interestingly, in juxtaposition with the review by Rogero et al. on inflammation in obesity, Dalton and colleagues report a study into systemic inflammation in individuals with the serious psychological eating disorder, anorexia nervosa [34]. They show that in a severely undernourished state, there are indications of systemic inflammation with an increased serum concentration of IL-6 when compared with healthy control participants. IL-6 is a classically inflammatory cytokine produced by immune and other cells. Whether this inflammation is the result of the impact of undernutrition or whether the clinical condition is the result of pre-existing inflammation is a matter that remains to be determined. It has been shown that patients with clinical depression have increased systemic inflammation suggesting that inflammation may have a bearing on mental health and wellbeing [35].

In contrast with the Western diet, the Mediterranean diet is rich in vegetables, fruit, nuts, legumes, fish, and ‘healthy’ dietary fats. The Mediterranean diet is associated with a reduced risk of chronic disease such as cardiovascular disease, cancer, and more recently Alzheimer’s disease [36]. A range of bioactive compounds found in fruits and vegetables have been reported to offer one explanation for the protective effect of diets rich in fruits and vegetables (e.g., Mediterranean diet) on the reduction of risk for developing non-communicable diseases attributed to chronic inflammation (e.g., cardiovascular disease). One family of molecules, which are known to have a role in regulation of inflammation are the dietary polyphenols [37]. Yahfoufi et al. explain the mechanisms by which polyphenols can be immunomodulatory and anti-inflammatory and explore the evidence for the role of dietary polyphenols in reducing the risk of cardiovascular disease, some neurological diseases, and cancer [38].

## 6. Nutrition in the Clinical Setting

In clinical settings, acute inflammation may be a sudden, severe, and overwhelming process. If not controlled, this severe systemic inflammation results in sepsis, culminating in multiple organ failure and death. Sepsis is a major global cause of death killing approximately 6 million people per year and is estimated to be the cause of 30% of neonatal deaths [39]. In this Special Issue of Nutrients, the role of zinc in sepsis is discussed [40]. Zinc is known to be an important micronutrient for the immune system. It has a role as a cofactor with both catalytic and structural roles in many proteins [41]. Even a mild deficiency in zinc has been associated with widespread defects in both the adaptive and innate immune response [42]. During sepsis, zinc homeostasis is profoundly altered with zinc moving from the serum into the liver. Alker and Haase consider this phenomenon and the implications for therapeutic options to improve outcomes in patients presenting with sepsis [40].

Selenium is a trace element that, like zinc, has critical functional, structural, and enzymatic roles, in a range of proteins. Poor selenium status is associated with a higher risk for range of chronic diseases including cancer and cardiovascular disease [43]. In addition to critical roles in many non-immune tissues within the body, selenium is important for optimal immune function. Avery and Hoffman explain the role of selenium in immunobiology and the mechanisms by which selenoproteins regulate immunity. The evidence for the significance of selenium status in infectious diseases including human immunodeficiency virus infection is reviewed [44].

Glutamine is a nonessential amino acid that provides an important energy source for many cell types including those involved in immune responses. It also serves as a precursor for nucleotide synthesis, particularly relevant for rapidly dividing cells such as the immune cells during an immune response. During infection, the rate of glutamine consumption by immune cells is equivalent or greater than that for glucose. Glutamine has roles in the functions of a number of immune cells including neutrophils, macrophages, and lymphocytes [45]. In catabolic conditions (e.g., infection, inflammation, trauma), glutamine is released into the circulation, an essential process controlled by metabolic organs such as the liver, gut, and skeletal muscles. Despite this adaptation, a significant depletion of glutamine is seen in the plasma and tissues in critical illness, which has provided a rationale for the use of in clinical nutrition supplementation of critically ill patients. How glutamine homeostasis is maintained and when and how to utilise glutamine in the clinical setting is explored in a review by Cruzat et al. [45].

The vitamin D receptor (VDR) is a nuclear receptor that can directly affect gene expression [46]. The presence of VDR in the majority of immune cells immediately suggests an important role for this micronutrient in immune cell activities [47]. Furthermore, vitamin D-activating enzyme 1-α-hydroxylase (CYP27B1), which results in the active metabolite 1 α,25-dihydroxyvitamin D_3_ (1,25(OH)_2_D_3_), is expressed in many types of immune cells. Ligation of VDR by 1,25(OH)_2_D_3_ can elicit the production of antimicrobial proteins and influence cytokine production by immune cells [47,48]. Sassi, Tamone, and d’Amelio have reviewed the evidence for the role of the nutrient vitamin D in the innate and adaptive immune systems [16].

## 7. Conclusions

In this Special Issue of Nutrients, the collected works provide a breadth of reviews and research indicating the key influence of nutrients and nutrition on immune responses in health and disease and across the life course. Nutrients may impact directly or indirectly upon immune cells causing changes in their function or may exert effects via changes in the gut microbiome. A better understanding of the role of nutrients in immune function will facilitate the use of bespoke nutrition to improve human health.
